# Study on energy evolution and fractal characteristics of sandstone with different fracture dip angles under uniaxial compression

**DOI:** 10.1038/s41598-024-60902-0

**Published:** 2024-05-07

**Authors:** Xin-yu Tian, Fu-jun Zhao, Wei-jun Wang, Biao Chen, Yu-jie Ma, Bao-jie Fan

**Affiliations:** 1 School of Resource and Environment and Safety Engineering, Hunan University of Science and Technology, Xiangtan, 411201 Hunan People’s Republic of China; 2 Changsha Institute of Mining Research Co. LTD, Changsha, 410017 Hunan China; 3 Urban Geological Survey and Monitor Institute of Hunan Province, Changsha, 410017 Hunan China

**Keywords:** Fracture inclination, Uniaxial compression, Strain energy, Energy feature, Fractal dimension, Engineering, Civil engineering

## Abstract

In order to investigate the failure modes and instability mechanism of fractured rock. Uniaxial compression tests were conducted on sandstone specimens with different dip angles. Based on rock energy dissipation theory and fractal theory, the energy evolution characteristics and fragmentation fractal characteristics in the process of deformation and failure of specimens were analyzed. The results show that the peak strength and elastic modulus of fractured rock mass are lower than those of intact samples, and both show an exponential increase with the increase of fracture dip angle. The energy evolution laws of different fracture specimens are roughly similar and can be classified into four stages based on the stress–strain curve: pressure-tight, elastic, plastic, and post-destructive. The total strain energy, elastic strain energy, and dissipated strain energy of the specimen at the peak stress point increased exponentially with crack inclination, and the dissipated strain energy and compressive strength conformed to a power function growth relationship. The distribution of the fragments after the failure of the fracture sample has fractal characteristics, and the fractal dimension increases with the increase of the fracture dip angle. In addition, the higher the compressive strength of the specimen, the greater the energy dissipation, the more serious the degree of fragmentation, and the greater the fractal dimension. The data fitting further shows that there is a power function relationship between the dissipated strain energy and the fractal dimension. The research results can provide a theoretical basis for the stability of rock mass engineering and structural deformation control.

## Introduction

Rocks that exist in nature are products of natural geological action and are filled with a variety of fissures that weaken the structural integrity and mechanical parameters of the rock mass. With the tunnelling, roadway excavation, and other underground works, the resulting disturbance effect makes the initial cracks inside the rock expand continuously, and interact with the subsequent sprouting cracks and penetrate to the rock damage, which has an important impact on the stability of rock engineering. In recent years, scholars at home and abroad have conducted a large number of experimental studies on the mechanical characteristics of fissured rocks. By conducting triaxial compression tests on non-penetrating fissure-like rocks with different inclination angles, Pu Cheng et al.^1^ found that the fissure inclination angle affects the post-peak plasticity properties of the specimens only within a certain range. Wu et al.^2^ explored the mechanical properties and crack evolution laws of prefabricated non-coplanar fractured rock specimens, and concluded that the prefabricated fracture penetration mode was tensile penetration and tensile-shear mixed penetration. Wu^3^ and Wu^4^ studied the influence of fracture number and dip angle on the mechanical properties of rock by numerical simulation test and laboratory test respectively. The results show that the strength of rock is mainly affected by the dip angle of fracture. Wang^5^, Xu^6^ and Wang^7^ explored the deformation and damage law of fissured rock under peripheral pressure, and concluded that the deformation and damage process of fissured rock can be divided into four stages, namely, crack germination, stable crack growth, and accelerated crack growth before and after the peak. Hao et al.^8^ carried out uniaxial compression tests on intersecting cracks with tips in dry and saturated states. The results show that the peak stress and initiation stress of the rock gradually increase with the increase of the angle between the intersecting cracks, and the existence of groundwater improves the lithology of the rock sample, and also weakens the stress concentration at the outer tip of the inclined crack. The above studies focused on the analysis of the mechanical properties during the deformation and destruction of fractured rock masses.

In the process of rock deformation and failure, the internal micro-cracks continue to initiate, expand and penetrate into the rock fracture. This process shows obvious fractal characteristics, and the fragmentation distribution of rock after fracture can indirectly reflect the meso-structure of rock. According to the law of thermodynamics, the essence of the deformation and failure process of rock is the dissipation and release of internal energy^9–13^, so there is a certain correlation between the law of rock energy dissipation and the fractal characteristics of fragmentation. Therefore, in order to further study the failure mechanism of rock, many scholars have analyzed the energy dissipation and fractal characteristics of rock fragmentation^14–17^. Zhang et al.^18^ analyzed the energy evolution law in the process of shale deformation and failure, and found that there was a quadratic nonlinear relationship between peak strain energy and compressive strength. Xie et al.^19^ conducted uniaxial compression and triaxial compression tests on sandstone containing fillings, and obtained that the dissipation ratio of the sample gradually increased with the increase of confining pressure. Liu et al.^20^ found that the failure mode of post-peak broken sandstone is related to energy absorption. Ji et al.^21^ and Yang et al.^22^ studied the energy dissipation and fragmentation fractal characteristics of rock under dynamic load, and found that there was a certain correlation between fractal dimension and energy dissipation. Yu et al.^23^ carried out uniaxial compression test on acid-corroded granite, and concluded that the dissipation energy decreased with the decrease of crushing degree. Zhang et al.^24^ found that the energy dissipation of sandstone under freeze–thaw cycles is linearly positively correlated with the fragmentation fractal. Li^25^ and Guo et al.^26^ conducted uniaxial compression tests of sandstone based on acoustic emission signals. It is considered that the acoustic emission signals increase significantly in the critical instability failure stage, and the cumulative location points and incremental location points of acoustic emission can characterize the whole process of rock damage and failure. The above scholars have made a lot of contributions to the study of energy mechanism and fragmentation fractal characteristics of various intact rock samples under different loading conditions, while the correlation between energy dissipation and fragmentation fractal characteristics of fractured rock mass is relatively scarce. Therefore, further research in this area can provide a theoretical basis for maintaining the stability of rock mass engineering.

In this paper, the uniaxial compression tests of red sandstone with different fracture dip angles are carried out. The influence of fracture dip angle on the mechanical properties of red sandstone is analyzed. The influence of fracture dip angle on the energy evolution and fractal dimension of sandstone in the process of deformation and failure is studied from the perspective of energy and fractal, and the mechanical mechanism between them is discussed, which provides a reference for elucidating the failure mechanism of rock.

## Test survey

### Testing specimens

**Figure 1:**
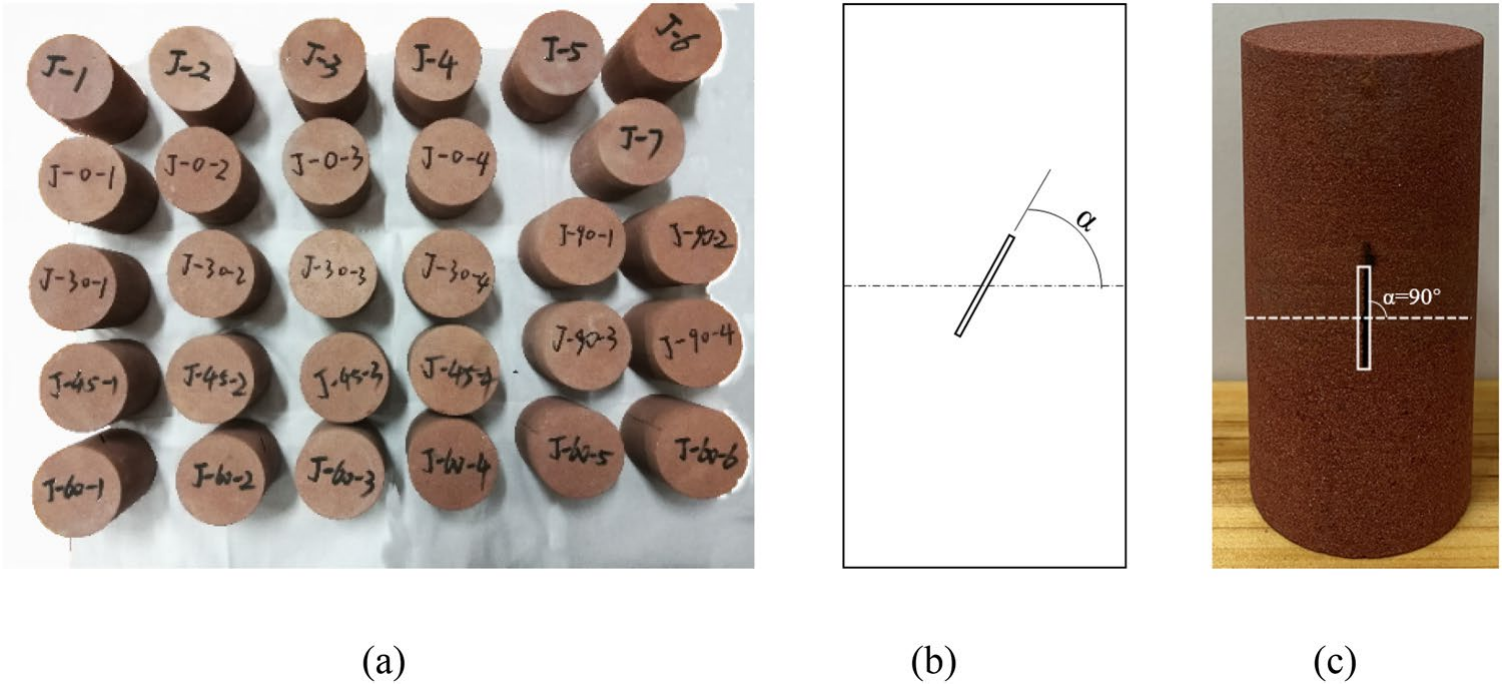
Schematic diagram of sample preparation: (**a**) all samples, (**b**) Schematic diagram of prefabricated crack angle, (**c**) Main view of specimen.

The red sandstone of a quarry in Sichuan province was selected as the research object for this test. To avoid the influence of rock anisotropy, specimens with the size of Φ50 × 100 mm were drilled from the same parent rock, and the specimen sections were polished with sandpaper so that the degree of non-parallelism and non-perpendicularity was lower than 0.02 mm. The prefabricated penetrating cracks with a length of 20 mm and a width of 1 mm were processed by high-speed water knife-cutting technology. The midpoint of the crack coincides with the center point of the specimen. The crack dip angle *α* is set to 0°, 30°, 45°, 60°, and 90°, respectively. There are two groups of samples: (1) complete samples. (2) Samples with different inclination angles of prefabricated cracks. (As shown in Fig. 1).

### Test device and test procedure

**Figure 2:**
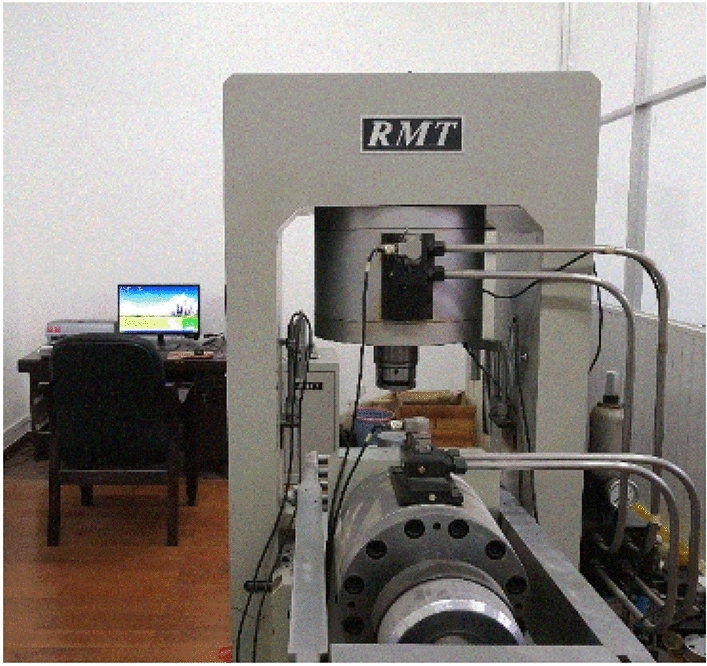
RMT-150C Rock Mechanics Test System.

This test uses the RMT150C rock mechanics test system (as shown in Fig. 2), which has the advantages of simple operation, high test accuracy, and good safety performance. The loading mode is displacement control, and the loading rate is 0.002 mm/s. The test parameters are input into the computer control system and preloaded. After the preload is completed, the test machine starts to pressure the sample until the sample is destroyed. The test machine supporting software automatically records the axial load and displacement data during the test.

## Test results and analysis

### Stress–strain curves

**Figure 3:**
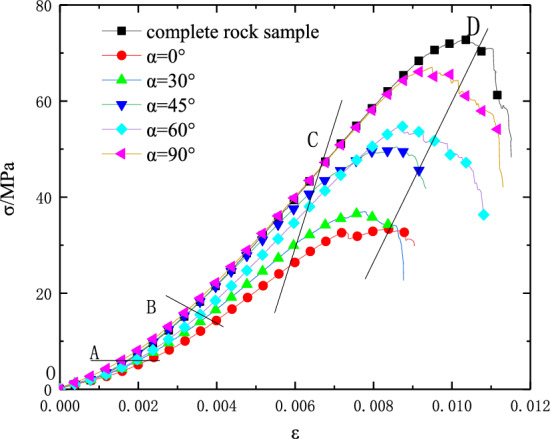
Stress and strain curves of intact and fractured rocks.

The complete stress–strain curves of the complete rock sample and rock samples with different prefabricated fissure dip angles are shown in Fig. 3. The uniaxial compression test results of intact and fractured rock are shown in Table 1. It can be seen from Fig. 3 that all specimens undergo a typical compaction stage (*OA* stage), elastic stage (*AC* stage), yield stage (*CD*), and post-failure stage (*D* later stage) from loading to failure. In the compaction stage, the original micro-cracks in the sample gradually closed under static load, and the stress–strain curve was nonlinearly concave. With the increase of load, the micro-cracks in the sample develop steadily into the elastic stage. The slope of the curve of the sample with different crack inclination angles is different, and the slope increases with the increase of the crack inclination angle. Before reaching yield point *C*, the stress–strain curve of the sample with the crack inclination angle of 90° is basically the same as that of the complete specimen, and there is no significant difference in the stress value. When loaded to the yield stress of the specimen, it changes from elastic deformation to plastic deformation, and the development of microcracks changes qualitatively. The cracks continue to develop and penetrate until the specimen is destroyed, reaching the peak strength, namely *D* point. In the post-failure stage, the specimen still has a certain bearing capacity, and then the specimen fragments slip and the stress–strain curve falls rapidly, all showing brittle failure characteristics.

**Table 1:** Test results of rock with complete and different fracture angles.

Fracture dip angle/°	Compressive strength		Elastic modulus	
	Numerical value/MPa	Decrease/%	Numerical value/GPa	Decrease/%
Complete sample	72.51	–	11.62	–
0	33.44	53.88	6.85	41.05
30	36.94	49.06	7.50	35.46
45	50.43	30.45	9.40	19.10
60	54.99	24.16	9.52	18.07
90	68.05	6.15	10.51	9.55

It can be seen from Table 1 that the compressive strength and elastic modulus of the complete sample are greater than those of the rock with prefabricated cracks, indicating that the crack dip angle has a significant effect on the strength and deformation parameters of the rock sample. The compressive strength of the intact sample and the samples with different crack inclination angles (0°, 30°, 45°, 60°, 90°) were 72.51 MPa, 33.44 MPa, 36.94 MPa, 50.43 MPa, 54.99 MPa, 68.05 MPa, respectively, which were degraded by 53.88%, 49.06%, 30.45%, 24.16%, and 6.15%, respectively. The modulus of elasticity of the intact specimens and crack inclination angles of 0°, 30°, 45°, 60°, and 90° were 11.62 GPa, 6.85 GPa, 7.50 GPa, 9.40 GPa, 9.52 GPa and 10.51 GPa, which were reduced by 41.05%, 35.46%, 19.10%, 18.07%, and 9.55%, respectively.

**Figure 4:**
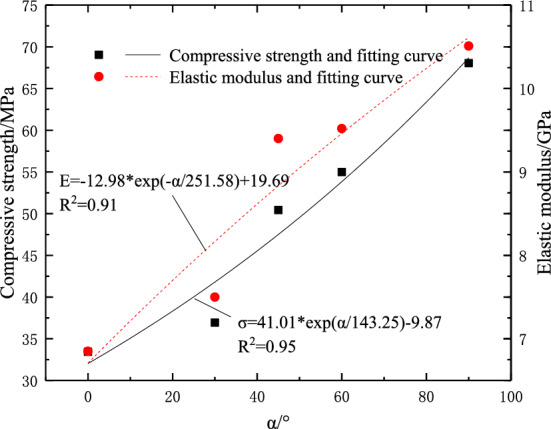
The variation curve of the peak strength and elastic modulus of the ample with the crack Angle.

The data fitting analysis of the fracture dip angle and mechanical parameters is shown in Fig. 4. It is found that the compressive strength and elastic modulus increase exponentially with the change of fracture dip angle. In the uniaxial compression test, the rock is only subjected to axial pressure, and the lateral deformation is not limited. Therefore, the microcracks generated during the compression process mainly propagate along the loading direction^27^. With the increase of load, the microcracks are approximately connected in the direction perpendicular to the loading direction. When the specimen contains prefabricated cracks perpendicular to the loading direction (i.e., the crack dip angle is 0°), the generated microcracks are more likely to interact through the prefabricated cracks so that the bearing capacity of the specimen decreases. With the increase of the crack dip angle, the tensile cracks parallel to the loading direction connected by the prefabricated cracks continue to decrease, so the compressive strength of the rock gradually increases.

### Destruction form

**Figure 5:**
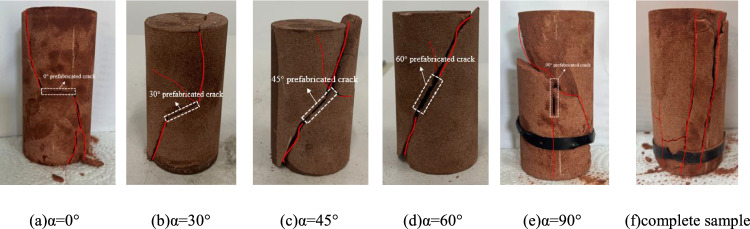
Failure modes of samples with different crack inclination angles.

The failure modes of intact specimens and specimens with different crack dip angles under uniaxial compression are shown in Fig. 5. The specimens with crack inclination angles of 0°, 30°, 45° and 60° all have a shear failure, which is manifested by stress concentration at the tip of the prefabricated crack, which initiates and produces wing-shaped shear cracks. With the increase of load, the wing-shaped shear cracks expand rapidly and penetrate each other until the specimen is destroyed (Fig. 5a–d). The specimen with a crack inclination angle of 90° belongs to tensile-shear failure. One of the tensile cracks extends along the lower end of the prefabricated crack to the lower part of the specimen, one of the shear cracks extends from the middle of the crack to the bottom, and the other shear crack extends from the upper part of the prefabricated crack to the side of the specimen (Fig. 5e). The failure mode of the complete sample is mainly splitting failure (Fig. 5f).

## Energy evolution

### Calculation method of strain energy

The whole process of rock failure is accompanied by energy input, energy accumulation, energy dissipation, and energy release^28^. According to the principle of thermodynamics, the failure of rock material is caused by its internal energy conversion. Assuming that in a closed system, the unit volume of rock material deforms under the action of external force, according to the first law of thermodynamics, energy conversion can be defined as:

1 $$ U = U_{{\text{e}}} + U_{d} $$

In the formula, *U* is the total strain energy, *U*_*e*_ is the elastic strain energy, and *U*_*d*_ is the dissipation energy.

The total strain energy accumulated inside the rock element in the principal stress space is:

2 $$ U = \int_{0}^{{\varepsilon_{1} }} {\sigma_{1} } {\text{d}}\varepsilon_{1} + \int_{0}^{{\varepsilon_{2} }} {\sigma_{2} } {\text{d}}\varepsilon_{2} + \int_{0}^{{\varepsilon_{3} }} {\sigma_{3} } {\text{d}}\varepsilon_{3} $$

In the formula, $\sigma_{1}$ and $\varepsilon_{1}$ are the first principal stress of rock element and its corresponding principal strain, respectively. $\sigma_{2}$ and $\varepsilon_{2}$ are the second principal stress of rock element and its corresponding principal strain, respectively. $\sigma_{3}$ and $\varepsilon_{3}$ are the third principal stress of rock element and its corresponding principal strain, respectively.

Under uniaxial compression load, there is $\sigma_{2} = \sigma_{3} = 0$, so Formula (2) can be rewritten as:

3 $$ U = \int_{0}^{{\varepsilon_{1} }} {\sigma_{1} } {\text{d}}\varepsilon_{1} $$

**Figure 6:**
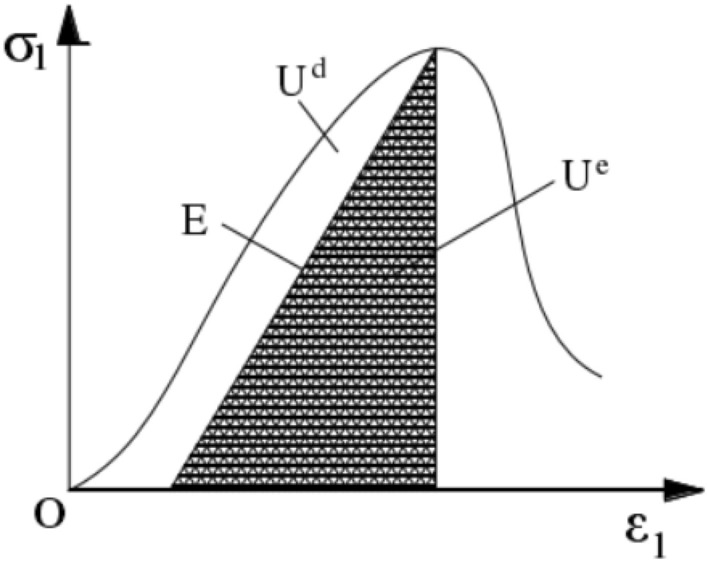
Relationship between dissipative strain energy *U*_*d*_ and releasable strain energy *U*_*e*_ of specimens^27^.

The total strain energy absorbed by the rock is converted into elastic strain energy produced by elastic deformation and dissipated strain energy produced by plastic deformation. The relationship between elastic strain energy *U*_*e*_ and dissipated strain energy *U*_*d*_ is shown in Fig. 6.

The elastic strain energy is:

4 $$ U_{e} = \frac{1}{2}\sigma_{1} \varepsilon_{1} = \frac{1}{2E}\sigma_{1}^{2} $$

In the formula, *E* is the unloading elastic modulus of rock. There is no cyclic loading and unloading test in this test. Therefore, the initial elastic modulus *E*_*0*_ can be taken as the unloading elastic modulus *E*. For the uniaxial compression test of rock, Formula (4) can be further simplified to^15^:

5 $$ U_{e} = \frac{1}{{2E_{0} }}\sigma_{1}^{2} $$

Bringing Formulas (3) and (5) into Formula (1), the dissipation energy can be expressed as:

6 $$ U_{d} = \int_{0}^{{\varepsilon_{1} }} {\sigma_{1} } {\text{d}}\varepsilon_{1} - \frac{1}{{2E_{0} }}\sigma_{1}^{2} $$

### Energy evolution characteristic curve

**Figure 7:**
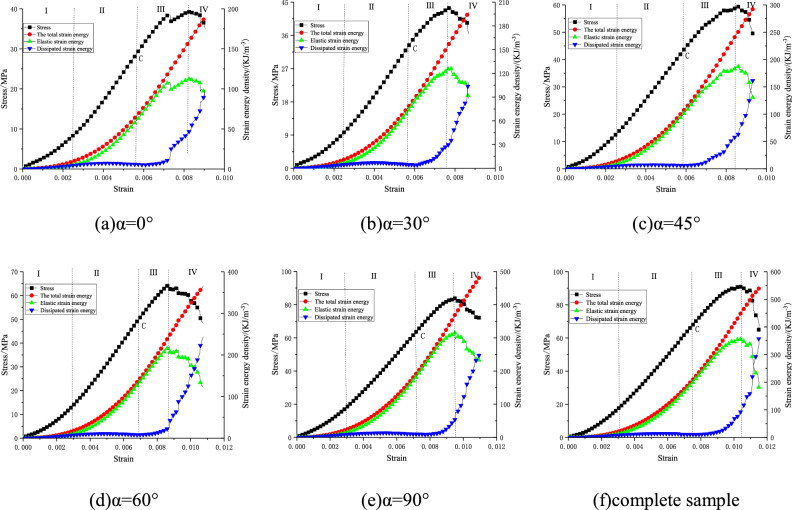
Stress–strain curve and strain energy evolution curve.

By using formulas (1)–(6), the total strain energy, elastic strain energy and dissipated strain energy in the process of deformation and failure of fractured rock mass are calculated, and the energy evolution curves in the process of deformation and failure of rock with different fracture dip angles are obtained, as shown in Fig. 7. It can be seen from the figure that the energy evolution law of specimens with different fracture dip angles is roughly similar, which corresponds to the four stages of stress–strain curve. Therefore, according to the stress–strain curve, the evolution process of strain energy is divided into four stages: initial compaction stage (stage I), elastic stage (stage II), plastic stage (stage III), and post-failure stage (stage IV).

1. Initial compaction stage (stage I): In the initial stage of loading, due to the small stress, the total strain energy, elastic strain energy, and dissipative strain energy increase very slowly. Before the closure of the original micro-cracks, the sample does not undergo elastic deformation. The energy in the input sample is mainly converted into dissipative strain energy, so the elastic strain energy in this stage is less than the dissipative strain energy.
2. Elastic stage (stage II): At this stage, the growth trend of total strain energy and elastic strain energy is consistent, which is greater than that of dissipative strain energy. Due to the closure of microcracks inside the specimen, the absorbed energy is mainly converted into elastic strain energy, and the variation curve of dissipated strain energy tends to be stable. However, as the load increases, the energy input into the rock sample further increases, and the elastic strain energy accumulated in the interior dissipates with the newly formed free surface energy, so the dissipated energy shows a convex upward trend.
3. Plastic stage (stage III): With the further increase of the load, the absorbed energy increases, the total strain energy still increases linearly, the growth rate of the elastic strain energy evolution curve gradually slows down, and the growth rate of the dissipative strain energy gradually increases until the stress reaches the peak point, the elastic strain energy reaches the peak, and the dissipative strain energy continues to grow. From Fig. 7a, it can be seen that the stress–strain curve of the sample falls downward, and the elastic strain energy characteristic curve also falls, while the dissipated strain energy increases suddenly at this time, indicating that when the prefabricated crack is completely compacted, part of the elastic energy is quickly released and converted into dissipated energy.
4. Post-failure stage (stage IV): When the sample reaches the peak strength, the elastic strain energy accumulated in the rock sample is rapidly released, and the curve shows a rapid decline, while the dissipation energy curve shows a rapid upward trend, and the total strain energy is no longer increased. As the micro-fracture of the specimen continues to develop, the cracks also expand and penetrate rapidly, and the dissipated strain energy continues to increase, resulting in complete failure of the specimen.

### Energy characteristics of peak stress point

Combined with the energy evolution curve, it can be seen that when the sample reaches the peak stress point, the elastic strain energy accumulated in the rock also reaches the peak value, that is, the energy storage limit of the sample reaches the peak value, and the elastic strain energy in the sample continues to be loaded. It will be converted into dissipative strain energy, and the dissipative energy will increase sharply. In addition, the greater the energy storage limit, the greater the total energy absorbed by the rock. In order to further study the energy relationship corresponding to the stress peak point of samples with different fracture dip angles, the energy indexes of samples with different fracture dip angles are listed in Table 2. It can be seen from Table 2 that the proportion of elastic strain energy of specimens with different fracture dip angles is 59.02%, 59.61%, 62.98%, 64.63%, and 68.36% respectively, and the proportion of dissipated strain energy is 40.98%, 40.39%, 37.02%, 35.37%, and 31.64% respectively. The elastic strain energy of the specimen at the peak stress point is greater than the dissipated strain energy, indicating that the total energy inside the pre-peak specimen is mostly converted to elastic strain energy, and the energy dissipation is small. In addition, compared with the 0° dip angle sample, the total strain energy increases by 3.59%, 55.20%, 71.12%, and 112.91% respectively with the increase of the dip angle of the fracture. With the increase of the dip angle, the initial damage caused by the fracture to the sample is reduced, and the ability of the sample to store the elastic strain energy is enhanced, so the total energy absorbed by the peak point rock gradually increases.

**Table 2:** Energy characteristics of peak stress points of samples with different crack inclination angles.

*α*/°	Total strain energy *U* (KJ·m^−3^)	Elastic strain energy *U*_*e*_		Dissipated strain energy *U*_*d*_	
		Numerical value (KJ·m^−3^)	Proportion/%	Numerical value (KJ·m^−3^)	Proportion/%
0	138.54	81.76	59.02	56.78	40.98
30	143.51	85.54	59.61	57.97	40.39
45	215.02	135.41	62.98	79.60	37.02
60	237.07	153.21	64.63	83.86	35.37
90	294.97	201.65	68.36	93.32	31.64
Complete sample	354.75	227.27	64.06	126.98	35.94

**Figure 8:**
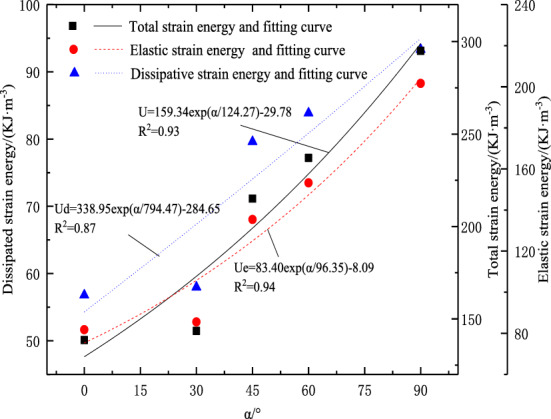
Variation curve of peak point energy index with crack Angle.

Figure 8 shows the relationship between the energy characteristics of the peak stress point and the fracture dip angle. It can be seen from the figure that the total strain energy, elastic strain energy, and dissipative strain energy of the sample increase with the increase of the fracture dip angle. The data fitting results show that the three are approximately exponentially related to the fracture dip angle, and the correlation coefficient is greater than 0.96, indicating that the fitting effect is good.

**Figure 9:**
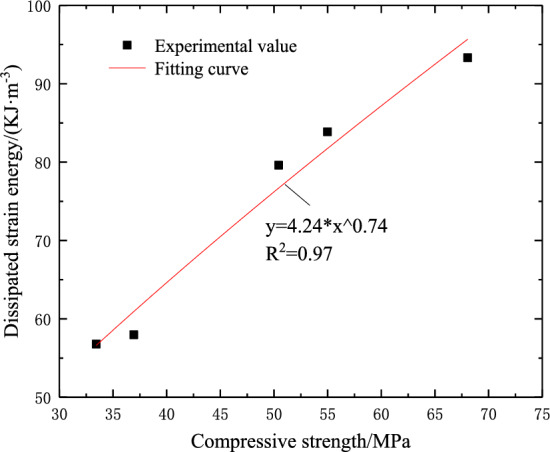
The relationship between dissipated strain energy and compressive strength.

In the process of uniaxial compression, the dissipated strain energy begins to increase significantly when the rock reaches the yield point. When the rock reaches the peak stress, the dissipated strain energy increases rapidly. Combined with Figs. 4 and 8, it can be seen that with the increase of fracture dip angle, the dissipated strain energy and compressive strength at the peak stress point of the sample show an increasing change rule. Therefore, there is a certain relationship between the two. Therefore, the data fitting of the dissipated strain energy and compressive strength at the peak stress point is carried out. The fitting results are shown in Fig. 9. It can be seen that the relationship between the dissipated strain energy and the compressive strength of the sample conforms to the following formula:

7 $$ U_{d} = 4.24\sigma^{0.74} ,R^{2} = 0.97 $$

It can be seen from Fig. 9 that the dissipation strain energy changes with the compressive strength in accordance with the power function growth trend. The higher the compressive strength of the sample, the greater the releasable elastic strain energy stored in the sample. When the sample is destroyed, the more the dissipated strain energy is converted, the higher the degree of damage.

## Fragmentation fractal characteristics

### Fractal dimension calculation method

To calculate the particle size and mass of rock debris after failure, the standard sieves with specifications of 0.074, 0.25, 0.5, 1, 2, 4, and 8 mm were used to screen the fragments after the test, and the fragments under the sieve were weighed. The particle size-mass statistical method was used to calculate the fractal dimension of the samples with different fracture dip angles. The distribution equation of rock fragmentation is:

8 $$ M(r)/M_{T} = (r/r_{m} )^{3 - D} $$

In the Formula: *M*_*T*_ is the total mass of debris; *r* is the particle size of the fragment; *M(r)* is the mass of debris with sieve diameter less than *r*; *r*_*m*_ is the maximum particle size; *D* is the fractal dimension of fragment distribution. By taking logarithms on both sides of Formula (8), we can obtain:

9 $$ \lg [M(r)/M_{T} ] = (3 - D)\lg (r/r_{m} ) $$

In the double logarithmic coordinate system, the least square method is used to fit the data, and the slope of the fitting line is (3-D), to obtain the fractal dimension *D* of the rock fragmentation distribution.

### Calculation results of fractal dimension

**Figure 10:**
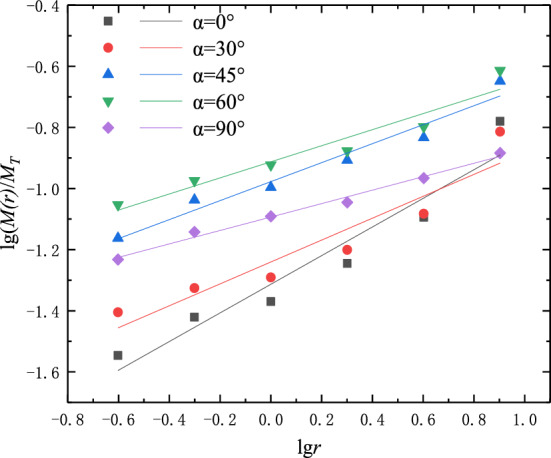
lg(M(r)/M)-lgr curves of samples with different crack angles.

Figure 10 shows the lg[*M(r)/MT*] and lg* r* curves of the fragmentation distribution of samples with different fracture dip angles. It can be seen from Fig. 10 that there is a good linear correlation between the double logarithm of the particle size-mass of each sample, and the correlation coefficients are all above 0.85, indicating that the distribution of broken blocks after compression failure of samples with different crack inclination angles has fractal characteristics. The higher the fractal dimension, the more the number of broken blocks of the sample, the smaller the particle size of the block, and the higher the degree of damage.

**Figure 11:**
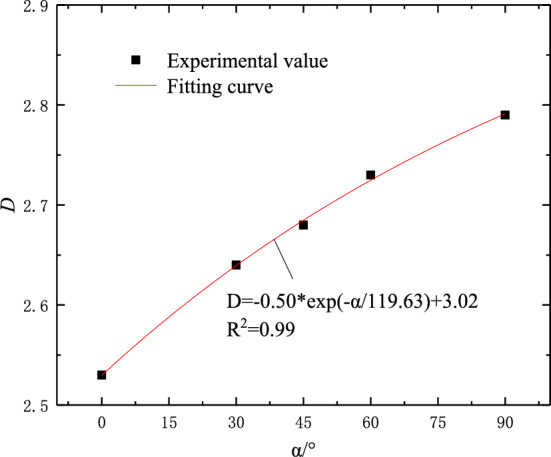
The relationship between fractal dimension and crack angles.

To analyze the influence of fracture dip angle on the fractal dimension of rock fragmentation, the relationship curve between the fractal dimension and fracture dip angle is drawn, as shown in Fig. 11. From the diagram, it can be seen that the fractal dimension is distributed between 2.50 and 2.80, and the data fitting analysis of the fractal dimension and the fracture dip angle is carried out. It is found that there is an approximate exponential growth relationship between the two, indicating that the larger the fracture dip angle, the deeper the damage and failure degree of the sample.

### Fractal dimension and compressive strength

**Figure 12:**
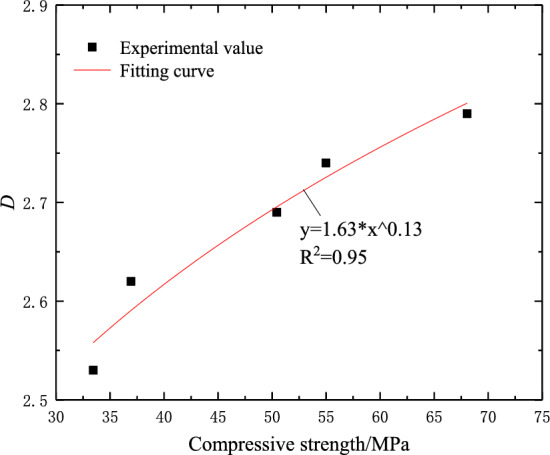
The relationship between the fractal dimension of the sample and the compressive strength.

Combined with Figs. 4 and 11, it can be seen that when the crack dip angle is small, the resistance to deformation of the sample is weak, and the sample is more prone to failure. There are fewer microcracks generated inside the uniaxial compression process, resulting in a lower degree of damage and a smaller fractal dimension. With the increase of fracture dip angle, the compressive strength increases, the micro-cracks generated during failure will increase significantly, the damage degree of the rock will be deeper, and the fractal dimension will also increase. The data fitting of fractal dimension and compressive strength is carried out. The fitting results are shown in Fig. 12. It can be seen that there is a power relationship between the fractal dimension and the compressive strength of the sample. The fitting function is:

10 $$ D = 1.63\sigma^{0.13} ,R^{2} = 0.95 $$

### Fractal dimension and dissipative strain energy

The above research shows that there is a positive correlation between the dissipated strain energy of the prefabricated crack specimen and the crack dip angle and compressive strength, and the correlation is good (see Figs. 8 and 9). The dissipated strain energy and the crack dip angle and compressive strength can be expressed as follows:

11 $$ U_{d} \propto \alpha \propto \sigma $$

From Figs. 11 and 12, it can be seen that there is also a positive correlation between the fractal dimension of fractured rock mass and the fracture dip angle and compressive strength, which can be expressed by the following formula:

12 $$ D \propto \alpha \propto \sigma $$

Combining Formulas (11) and (12), it can be concluded that there is also a positive correlation between the fractal dimension of the sample and the dissipated strain energy. With the increase of the fracture dip angle, the stronger the resistance to deformation of the sample, the more strain energy consumed by crack initiation, propagation, and coalescence during rock failure, resulting in a higher degree of fragmentation and more small debris scales, so the fractal dimension is larger. The relationship between the two can be expressed by the following formula:

13 $$ U_{d} \propto D $$

**Figure 13:**
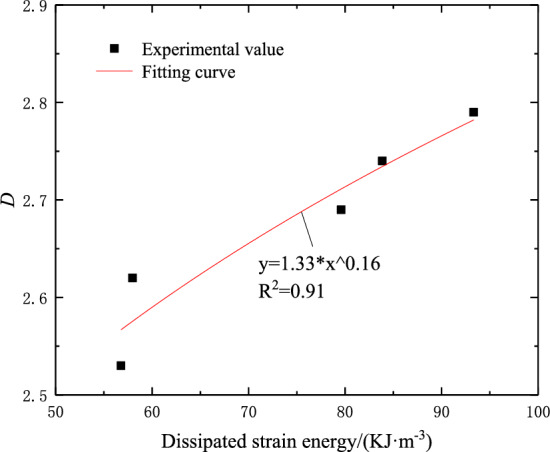
The relationship between the fractal dimension of the sample and the dissipated strain energy.

The data fitting analysis of fractal dimension and dissipative strain energy is carried out. The fitting results are shown in Fig. 13. It is found that the fractal dimension of the sample increases with the increase of the dissipative strain energy. The fitting curve conforms to the power relationship, and the fitting formula is shown in Eq. (14).

14 $$ D = 1.33U_{d}^{0.16} ,R^{2} = 0.91 $$

## Conclusion

1. The stress–strain curves of intact rock and specimens with prefabricated cracks have gone through the same stage. With the increase of dip angle, the compressive strength and elastic modulus of fractured rock increase exponentially, but they are lower than those of intact samples. The failure mode shows the change from shear failure to tensile-shear composite failure and splitting failure.
2. The energy evolution law of specimens with different fracture dip angles is roughly similar, and there is a certain corresponding relationship with the stress–strain curve, which can be divided into compaction stage, elastic stage, plastic stage and post-failure stage. At the peak stress point, with the increase of fracture dip angle, the total strain energy, elastic strain energy, and dissipated strain energy all increase exponentially, and the dissipated strain energy and compressive strength conform to the power function growth relationship.
3. The distribution of rock fragments after fragmentation has good fractal characteristics, and the fractal dimension increases exponentially with the increase of fracture dip angle.
4. With the increase of fracture dip angle, the stronger the resistance to deformation of the specimen, the greater the energy storage limit, the greater the dissipated energy when failure occurs, and the more obvious the fractal characteristics of fracture. Further data fitting of dissipative strain energy and fractal dimension shows that there is a power function relationship between them.

## Data Availability

All the generated and analyzed data are available from the corresponding author upon request.
